# Topiramate-Induced Modulation of Hepatic Molecular Mechanisms: An Aspect for Its Anti-Insulin Resistant Effect

**DOI:** 10.1371/journal.pone.0037757

**Published:** 2012-05-23

**Authors:** Hanan S. El-Abhar, Mona F. Schaalan

**Affiliations:** 1 Department of Pharmacology, Faculty of Pharmacy, Cairo University, Cairo, Egypt; 2 Department of Biochemistry, Faculty of Pharmacy, Misr International University (MIU), Cairo, Egypt; University of Colorado Denver, United States of America

## Abstract

Topiramate is an antiepileptic drug known to ameliorate insulin resistance besides reducing body weight. Albeit liver plays a fundamental role in regulation of overall insulin resistance, yet the effect of topiramate on this organ is controversial and is not fully investigated. The current work aimed to study the potential hepatic molecular mechanistic cassette of the anti-insulin resistance effect of topiramate. To this end, male Wistar rats were fed high fat/high fructose diet (HFFD) for 10 weeks to induce obese, insulin resistant, hyperglycemic animals, but with no overt diabetes. Two HFFD-groups received oral topiramate, 40 or 100 mg/kg, for two weeks. Topiramate, on the hepatic molecular level, has opposed the high fat/high fructose diet effect, where it significantly increased adiponectin receptors, GLUT2, and tyrosine kinase activity, while decreased insulin receptor isoforms. Besides, it improved the altered glucose homeostasis and lipid profile, lowered the ALT level, caused subtle, yet significant decrease in TNF-α, and boosted adiponectin in a dose dependent manner. Moreover, topiramate decreased liver weight/, visceral fat weight/, and epididymal fat weight/body weight ratios. The study proved that insulin-resistance has an effect on hepatic molecular level and that the topiramate-mediated insulin sensitivity is ensued partly by modulation of hepatic insulin receptor isoforms, activation of tyrosine kinase, induction of GLUT2 and elevation of adiponectin receptors, as well as their ligand, adiponectin, besides its known improving effect on glucose tolerance and lipid homeostasis.

## Introduction

Westernized diet, prevalent in modern society, is characterized by the increased consumption of high fat diet (HFD) along with fructose as a common sweetener. This type of food, together with the slothful lifestyle, participates in the amplification of the global wave of obesity, which is invariably associated with a constellation of pathological perturbations, among which is insulin resistance (IR) [1]. IR is manifested by the failure of endogenous insulin to hinder hepatic gluconeogenesis and to induce glucose peripheral uptake. Such dysfunction is compensated by hyperinsulinemia, and associated with alteration in glucose and lipid homeostasis, with or without overt diabetes [2]. Moreover, this type of diet frequently induces hepatic steatosis, which accounts for hepatic insulin resistance [3], and favors muscular fat storage, as well as visceral fat deposition [1], facilitating thus, the development of peripheral insulin resistance [4]. In addition, HFD-induced increased white adipose tissue (WAT) participates in the induction of whole body insulin resistance, via increasing the secretion of the pro-inflammatory cytokine, TNF-α, which impairs insulin signaling [5]. On the other hand, adiponectin, another adipohormone secreted also by adipocytes, is present in low concentration in obesity-insulin resistant states [6]. Furthermore, fructose, the main sweetener of westernized diet, is deemed to be a contributing factor to these epidemic disorders [7], where exposure of liver to such large quantities of fructose and fats leads to rapid stimulation of lipogenesis with the accumulation of triglycerides (TGs) that contribute, in turn, to reduced insulin sensitivity and hepatic insulin resistance/glucose intolerance [8].

Peripheral insulin resistance emanates from interplay between three major organs, viz., liver, adipose tissue and skeletal muscles, with altered insulin signaling that stems from altered insulin receptors and post-receptor defects at the molecular level. These include alterations in the relative abundance of the two insulin receptor isoforms, decrease in insulin affinity to its receptors, improper insulin receptor kinase activity, decrease in autophosphorylation, and abnormalities in glucose transporter translocation and activation [9].

Topiramate (TPM), a sulfamate-substituted monosaccharide, is a neurotherapeutic agent currently indicated for the management of epilepsy and migraine, and is documented to possess weight reducing properties, as well as insulin resistance improving effects [10]. Moreover, TPM has been reported to lower TGs and circulating FFAs, and to increase adiponectin levels [11]. Albeit liver is a cornerstone in the maintenance of insulin sensitivity and glucose metabolism, yet most studies on TPM were much more concerned with studying the effects on skeletal muscles and adipose tissue. Hence, the goal of this study was to enhance our understanding of the possible hepatic mechanisms related to insulin sensitivity and its modulation by TPM. To this end, a murine obese-insulin resistant model, but not with overt diabetes, was adopted to evaluate the effect of TPM on some hepatic molecular elements, besides the usual glucose/lipid homeostasis parameters, as well as the levels of the two contradictory cytokines, viz., adiponectin and TNF- α.

## Materials and Methods

### 1. Drugs and Chemicals

Topiramate (Topamax) was purchased from Janssen Cilag Co. (AG, Schaffhausen, Switzerland), and fructose was obtained from El-Nasr Chemical-Co. (Abou Zaabal, Cairo, Egypt). Cholesterol and lard were obtained from commercial sources and were of analytical grades.

### 2. Animals

Adult male Wistar rats (80–120 g) were purchased from National Research Center Laboratory (Cairo, Egypt). Rats were singly caged with free access to water and commercially available normal rat pellet diet prior to dietary manipulation. Animals were kept on a 12 hr light/dark cycle, and constant environmental conditions. Experimental design and animal handling were approved by the Research Ethical Committee of the Faculty of Pharmacy, Cairo University, Cairo, Egypt.

### 3. Development of Obese Insulin-resistant Rats

After three days of acclimatization, fifty rats were divided into two dietary regimen-groups, normal fat diet (NFD, n = 10) [3150 cal/g; fat (5%), protein (21%), carbohydrate as starch (60%), fibers (3%), vitamins and minerals (1%)], and high fat/high fructose diet (HFFD, n = 40) [5300 cal/g; fat (15% composed of 10% as lard and 1% cholesterol powder), protein (21%), carbohydrate as fructose (60%), fibers (3%), vitamins and minerals (1%) [12]. Diet manipulation lasted for 10 weeks until significant weight gain and insulin resistance were achieved. Animals were monitored once a week for weight gain along with alterations in fasting serum glucose, triglyceride (TG), and total cholesterol (TC). After 10 weeks, insulin level and glucose tolerance test (GTT) were undertaken, and only rats with increased weight gain, impaired glucose tolerance, hyperinsulinemia, hypertriglyceridemia, and hypercholesterolemia were considered obese/insulin-resistant and were used in the study.

### 4. Intra-peritoneal Glucose Tolerance Test (IPGTT)

The test was performed to confirm the state of insulin resistance, where after six hours of fasting animals from both groups, viz., NFD, and HFFD, were injected by glucose (2 g/kg, i.p). Droplets of blood from the tail vein were then drawn at 0 (prior to glucose load), 30, 60, 90 and 120 minutes (after glucose load) for glucose assay. AUC was calculated for blood glucose during the IPGTT according to the following equation: AUC = 0.25×(fasting value)+0.5×(1/2 h value)+0.75×(1 h value)+0.5×(2 h–value) [13].

### 5. Experimental Design

Rats with confirmed IR (n = 30) were further subdivided into three groups, two of them were given daily oral doses of either TPM 40 mg/kg ([14], HFFD/TPM_40_), or TPM 100 mg/kg ([15], HFFD/TPM_100_) for 2 weeks. The last 10 IR rats received saline and served as positive control group, while the NFD fed rats represented the negative normal control group and received saline, as well. Pair feeding was implemented to control for the food restriction effects of TPM. Pair-fed rats consumed equal portions of food as TPM-treated rats daily, ranging from 13–15 g/day.

All four groups continued their indicated diet till the end of experiment and the last dose of any treatment was given 24 hours before killing the rats, which were fasted 18 hours before the time of carnage, to minimize feeding induced variations in lipid and glucose pattern.

To exclude the possible effect of both dose levels of TPM (40 and 100 mg/kg) on the negative normal control rats (NFD), a preliminary study was performed and the assessed parameters are reported in Table 1.

**Table 1:** Effect of topiramate on serum and tissue biomarkers of normal control rat.

	Normal Control (NFD+saline)	Normal Control +TPM_40_	Normal Control +TPM_100_
Glucose (mg/dl)	82±7.1	85±6	86±8
Insulin (µIU/ml)	14.5±3	16.2±3	16.8±4
HOMA-index	2.97±0.2	3±0.5	3.2±0.4
Fructosamine (nmol/L)	129.4±11.6	135.2±12	137.5±13
AUC	229±22	249±15	219±16
TG (mg/dl)	42±4	44±4	45±3
TC (mg/dl)	61±5.4	63±6	59±5
FFAs (mmol/L)	0.79±0.08	0.72±0.08	0.82±0.1
ALT (IU/L)	40±5	42±3	39±4
TNF-α (pg/ml)	11±1.5	12±1.6	10.5±0.9
Adiponectin(ng/ml)	1.4±0.2	1.5±0.3	1.6±0.5
BW (g)	250±20	240±13	232±20
LW (g)/BWx100	2.5±0.15	2.4±0.2	2.4±0.3
VFW (g)/BWx100	0.73±0.03	0.75±0.03	0.72±0.02
EFW (g)/BWx100	0.35±0.04	0.37±0.03	0.3±0.02

Effect of 14 days administration of topiramate (40, 100 mg/kg) to normal control rats fed normal diet. The following markers were assessed: serum levels of glucose, insulin, and fructosamine, HOMA-index and area under the curve (AUC); serum triglycerides (TGs), total cholesterol (TC), free fatty acids (FFAs), ALT (IU/L), tumor necrosis alpha (TNF-α) and adiponectin; body weight (BW) and liver weight (LW)/−, visceral fat weight (VFW)/− and epididymal fat weight (EFW)/−BW ratio. Values are means (± S.D.) of 10 animals. Treatments were administered once daily for 2 weeks. Statistical analysis between groups was carried out using one-way ANOVA followed by Tukey-Kramer Test, *P*<0.05.

### 6. Collection of Serum Samples for Analysis

At the time of carnage, animals were weighed and blood was collected from the tail vein under brief ether anesthesia, and centrifuged (800×g, 4°C, 20 min) to separate sera. Animals were then sacrificed by cervical dislocation and liver, visceral fat and epididymal fat were carefully dissected out and weighed. Sera were used to determine glucose [16], fructosamine [17], TGs [18], TC [19], and ALT using commercially available Randox colorimetric reagent kits (Antrim, U.K). Insulin and adiponectin levels were estimated using RIA kit (Linco Research, St Charles, MO,USA) [20], while TNF-α was measured using ELISA kit (R&D systems, Minneapolis, USA). Homeostasis Model Assessment-index (HOMA-index) was calculated according to the following equation: [(glucose concentration (mmol/L) × Insulin (µU/L)/22.5] [21].

### 7. Tissue Weights

The changes in body weight (BW) were monitored every week, and the liver weight (LW), visceral fat weight (VFW) and epididymal fat weight (EFW) were expressed as a ratio of body weight (BW) multiplied by a factor of 100.

### 8. Tissue Extracts

For the following assays livers were rinsed in ice-cold phosphate buffer saline (PBS, 0.02 mol/l, pH 7.0–7.2) to remove excess blood thoroughly and weighed before homogenization. The minced liver tissues (0.5 g) were homogenized in 5 ml of PBS and the resulting suspension was subjected to two freeze-thaw cycles to break the cell membrane then sonicated with an ultrasonic cell disrupter. Afterwards, homogenates were centrifuged for 5 minutes at 5000×g and the supernatant was collected, measured and immediately divided into aliquots and stored at −20°C for the ELISA assay of adiponectin receptor R1and R2 (Uscn Life Science Inc. Wuhan, China), and GLUT2 (glucose transporter 2 ELISA kit,Wuhan, China).

#### 8.1. Detection range for adiponectin R1and R2

For adiponectin R1the standard curve concentrations used for ELISA were 10, 5, 2.5, 1.25, 0.625, 0.312, and 0.156 ng/mL, while those of adiponectin R2 were 20, 10, 5, 2.5, 1.25, 0.625, and 0.312 ng/mL.

#### 8.2. Determination of tyrosine kinase activity

Dissected liver tissues, after determination of the protein content to contain 1–5×10^6^ cells, were washed with PBS and spun at room temperature for 5 min. at 300×g and then the cells were recovered as pellets. One ml of extraction buffer was added to each sample, and the pellet was then suspended by vortex gently. The supernatant was recovered as the samples by spinning at 4°C for 10 min. at 10,000×g and remained stable at - 80°C for few days. The enzymatic activity was assessed according to the procedures stated by the Universal Tyrosine Kinase Assay Kit (TAKARA BIO INC., Tokyo, Japan).The activity of enzyme is based on the activity of recombinant c-Src, where one unit (U) of the enzyme is defined as the amount needed to incorporate 1 pmol of phosphate into the substrate in 1 min. The activity of protein tyrosine kinase (PTK) control in this kit is shown as the activity of c-Src. The activity of PTK is determined by comparing its absorbance with that of the PTK standard supplied in the kit. The measuring range of the kit lies between 32 fmol/well to 2 pmol/well (from 2.16×10^−5^ U/µL sample to 135×10^−5^ U/µl sample). The detection sensitivity is 2.16×10^−5^ U/µl and the measurement range is from 2.16×10^−5^−135×10^−5^ U/µL (86.4×10^−5^ to 5,400×10^−5^ U/well).

#### 8.3. Protein assay

The protein content was estimated by Lowry et al. [22] method using bovine serum albumin as standard.

#### 8.4. Assessment of insulin receptors LAIR and HAIR

The insulin receptor isoforms, viz., high affinity insulin receptor (HAIR) and low affinity insulin receptor (LAIR), were assessed using HPLC. Standards for HAIR (fmol/L, standard unit) and LAIR (pmol/L, standard unit) were purchased from Sigma-Aldrich Company (St. Louis, MO, USA). The chromatographic conditions were adjusted as follows: the mobile phase consists of sodium acetate buffer: acetonitrile in the ratio of 73∶27. The buffer was prepared by dissolving 3.0 g of sodium acetate in 500 ml of HPLC grade water and the pH was adjusted at 5. The mobile phase was pumped at a flow rate of 1.0 ml/min. The column was maintained at 45°C and the volume of each injection was 20 µl. Prior to solutions’ injection, the column was equilibrated for at least 20 min with mobile phase flowing through the system. The eluents were monitored at 243 nm, using Beckman gradient liquid chromate-graph with a dual pump system comprising: Model 126 AA pump, Model 210 sample injection valve: A Rheodyne 20 µl stainless steel loop injection and Microprocessor system controller. The HPLC column is an octadecylsilane (C18) reversed phase analytical partition column (Alltech^,^s, 150 mm long×4.6 mm internal diameter, 5.0 µm particle size) and the detector: Model 166- Beckman UV-Visible spectrophotometer.

### 9. Statistical Analysis

Results are expressed as means ± SD of 10 animals, and differences between groups were tested for significance using analysis of variance (ANOVA), followed by Tukey-Kramer *post hoc* test. Correlation coefficient (r) between HOMA-index and adiponectin with hepatic adiponectin receptors, insulin receptor isoforms, protein kinase activity and GLUT2, was carried out in untreated and treated insulin resistant animals using linear regression analysis. The level of statistical significance was taken at *P*<0.05.

## Results

Table 1 reveals that the normal control rats, administered TPM at both dose levels (40 and 100 mg/kg), showed no significant difference compared to the normal control group receiving saline. Therefore, the normal control group, NFD, was chosen for all statistical comparisons.

**Figure 1:**
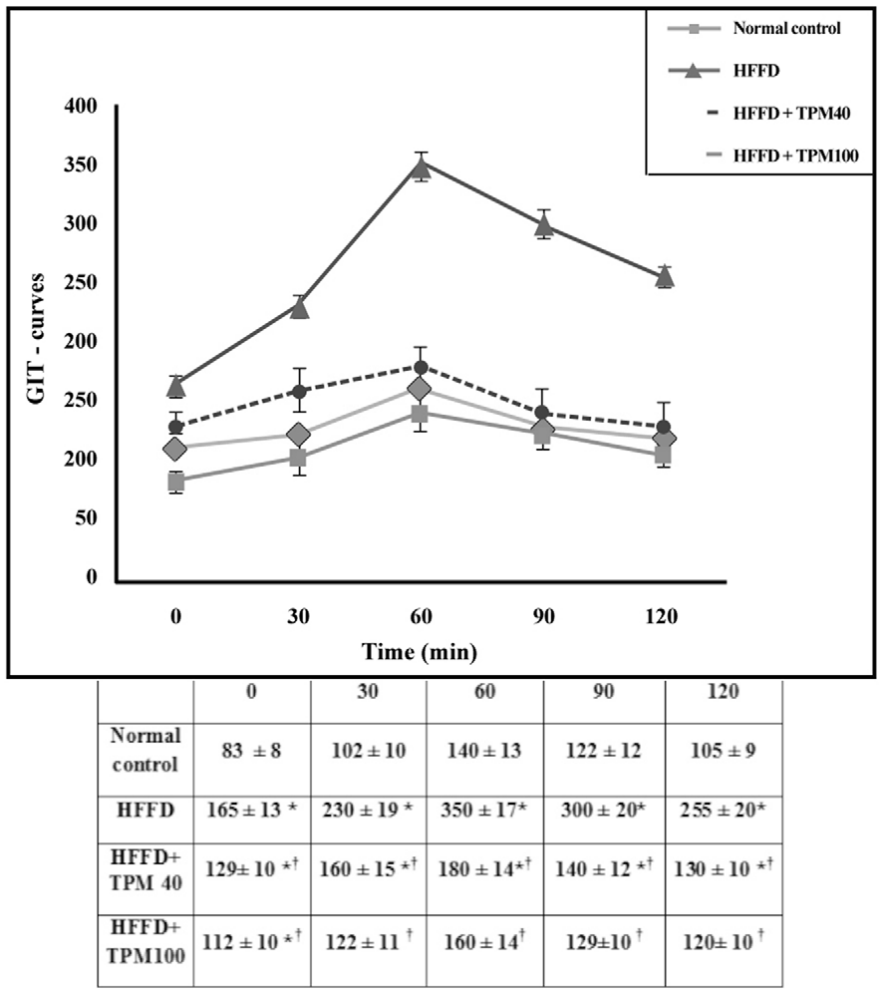
The glucose tolerance test (GTT). The curve depicts the changes in serum glucose response in normal control, non-treated obese/insulin-resistant rats (HFFD), and treated ones by either dose of topiramate (40 & 100 mg/kg;HFFD+TPM40, HFFD+TPM_100_), after 0, 30, 60, 90, and 120 min following administration of glucose (2 g/kg, ip). Values are means (± S.D) of 10 animals; as compared with normal control (^*^), and HFFD (**^†^**) groups (one-way ANOVA followed by Tukey–Kramer Test), *P*<0.05.

**Figure 2:**
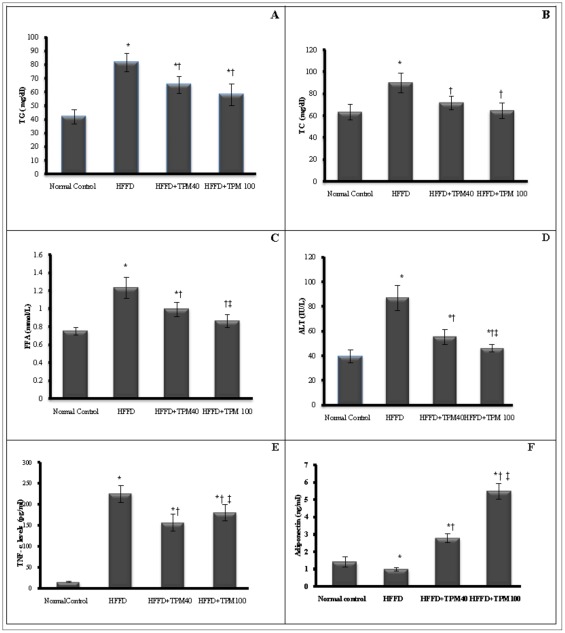
Effect of topiramate on serum biomarkers. The panels illustrate the effect of topiramate (40 & 100 mg/kg, p.o; TPM_40_, TPM_100_) on serum (A) triglycerides (TGs, [mg/dl]), (B) total cholesterol(TC, [mg/dl]), (C) free fatty acids (FFAs, mmol/l]), (D)ALT (IU/L), (E) tumor necrosis alpha (TNF-α, [pg/ml]) and (F) adiponectin [ng/ml] of obese/insulin resistant rats fed high fat and high fructose diet [HFFD] for 10 weeks (mean of 10 animals ± S.D.). Treatments were administered once daily for 2 weeks. As compared with normal control (^*^), HFFD (**^†^**) and HFFD+TPM40 ^(**‡**)^ groups (one-way ANOVA followed by Tukey–Kramer Test), *P*<0.05.

Figure 1 depicts the IPGTT performed in untreated HFFD, topiramate (40 and 100 mg/kg) treated HFFD rats, and normal control ones. Compared to the normal control group, HFFD diet elevated serum glucose level markedly after glucose injection, an effect that leveled off significantly after treatment with either dose of TPM; these alterations in GTT were imitated by the AUC values (Table 2). Topiramate treated groups also opposed the HFFD-induced alterations in insulin-glucose panel as presented in Table 2, and corrected the lipid profile in a similar pattern (Figures 2A-C), as well as the ALT level (Figure2-D). As shown in Figure 2 (E, F), HFFD boosted TNF-α level (14.8 times), but decreased that of adiponectin (30.4%), respectively. Both TPM_40_ and TPM_100_ inhibited the TNF-α level significantly by 30 and 20%, respectively), while raised that of adiponectin, above the normal level by 2.8 times and 5.6 times, respectively.

**Table 2:** Effect of topiramate on the indicators of glucose homeostasis of obese/insulin resistant rats.

	Glucose (mg/dl)	Insulin (µIU/ml)	HOMA-index	Fructosamine (nmol/L)	AUC
Normal Control	82±7.1	14.5±3	2.97±0.2	129.4±11.6	229±22
HFFD	165±13^*^	35.1±2 ^*^	14.47±1.3^*^	191.3±13.5 ^*^	546±43.0*
HFFD+TPM_40_	128±11^*†^	17.8±2 ^†^	5.69±0.4^*†^	135.4±12.5 ^†^	312±24^*†^
HFFD+TPM_100_	112±8^*†‡^	15.6±3 ^†^	4.36±0.3^*†‡^	136.8±14 ^†^	269±24.9^†^

Effect of topiramate (40, 100 mg/kg; TPM_40_, TPM_100_) on serum levels of glucose, insulin, and fructosamine, HOMA-index and area under the curve of the GTT of obese/insulin resistant rats fed high fat and high fructose diet [HFFD] for 10 weeks. Values are means (± S.D.) of 10 animals. Treatments were administered once daily for 2 weeks. As compared with normal control (^*^), HFFD (**^†^**) and HFFD+TPM_40_ **(^‡^)** groups (one-way ANOVA followed by Tukey–Kramer Test), *P*<0.05.

HFFD also increased BW, as well as LW/−, VFW/−, and EFW/− BW ratios. Albeit TPM_100_ significantly leveled off all these ratios, yet it unexpectedly did not affect body weight (Table 3).

**Table 3:** Effect of topiramate on body-, liver- and fat weights of obese/insulin resistant rats.

	BW (g)	LW/BW x100	VFW/BWx100	EFW/BWx100
Normal Control	250±20	2.5±0.15	0.73±0.03	0.35±0.04
HFFD group	348±35^*^	3.18±1.27^*^	1.01±0.09^*^	0.78±0.08^*^
HFFD+TPM_40_	347.5±37^*^	2.90±0.2^*^	0.85±0.1	0.68±0.07^*^
HFFD+TPM_100_	325±40^*^	2.69±0.2^†^	0.78±0.04^†^	0.56±0.06 ^†^

Effect of topiramate (40, 100 mg/kg; TPM_40_, TPM_100_) on body weight (BW) and liver weight (LW)/−, visceral fat weight (VFW)/− and epididymal fat weight (EFW)/−BW ratio of obese/insulin resistant rats fed high fat and high fructose diet [HFFD] for 10 weeks. Values are means (± S.D.) of 10 animals. Treatments were administered once daily for 2 weeks. As compared with normal control (^*^), and HFFD (**^†^**) groups (one-way ANOVA followed by Tukey-Kramer Test), *P*<0.05.

**Figure 3:**
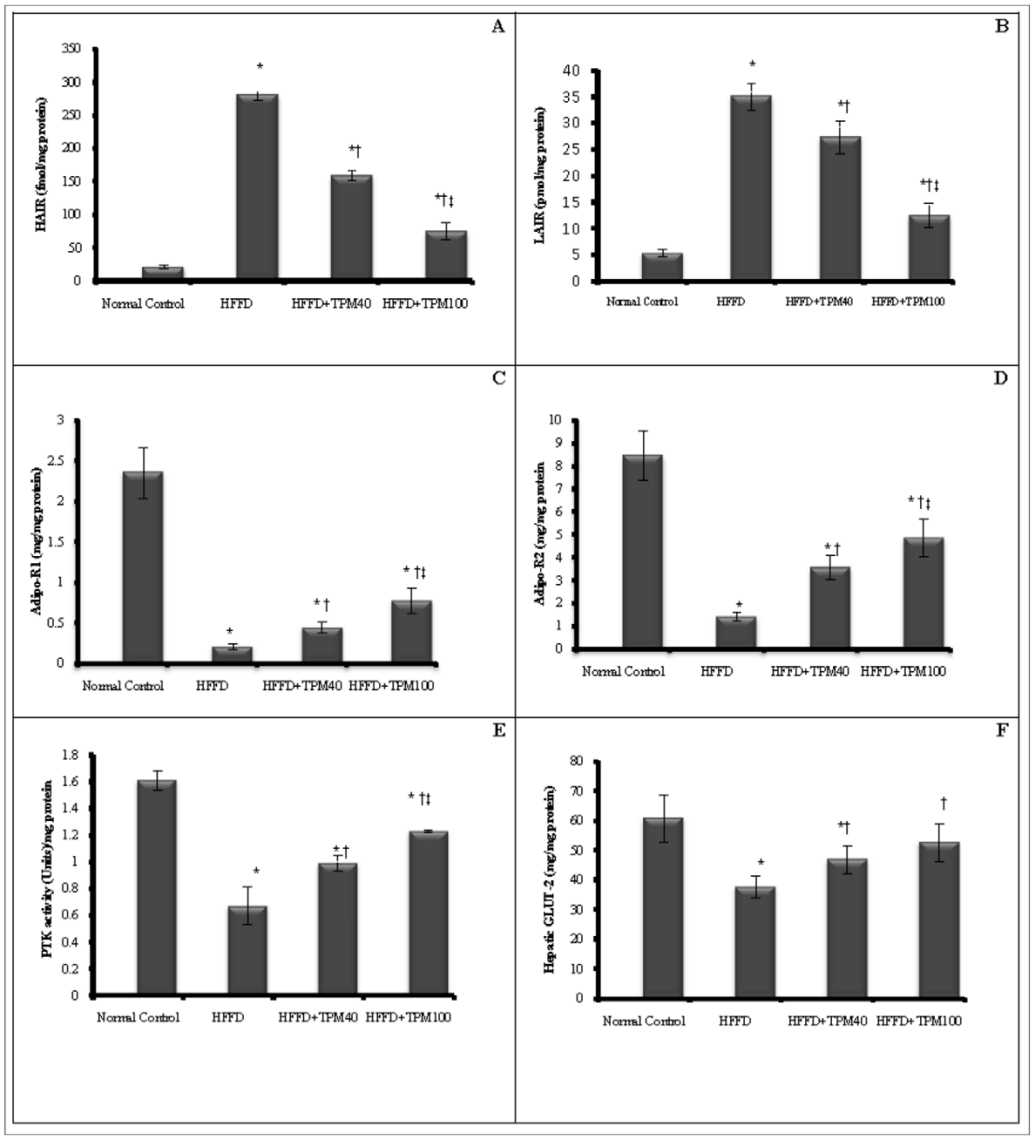
Effect of topiramate on hepatic biomarkers. The panels illustrate the effect of topiramate (40 & 100 mg/kg, p.o; TPM_40_, TPM_100_) on hepatic (A, B) insulin receptor isoforms (high affinity, [HAIR, fmol/mg protein] and low affinity [LAIR, pmol/mg protein] insulin receptor), (C, D) adiponectin receptors (Adipo-R1, Adipo-R2 [ng/mg protein]), (E) protein tyrosine kinase (PTK [U/mg protein]), and (F) glucose transporter-2 (GLUT2 [mg/mg protein]) of obese/insulin resistant rats fed high fat and high fructose diet [HFFD] for 10 weeks (mean of 10 animals ± S.D.). Treatments were administered once daily for 2 weeks. As compared with normal control (^*^), HFFD (**^†^**) and HFFD+TPM40 ^(‡)^ groups (one-way ANOVA followed by Tukey–Kramer Test), *P*<0.05.

Regarding the hepatic molecular parameters, HFFD increased the abundance of the two insulin receptor isoforms (Figure 3 A, B), while significantly lowered those of adiponectin (Figure 3 C, D), as well as tyrosine kinase activity (Figure 3E) and GLUT2 level (Figure 3F). The two doses of TPM hampered the HFFD effect on all these parameters; however, the higher dose showed a more pronounced effect. Using linear regression analysis, HOMA-index correlated negatively with hepatic Adipo-R1, Adipo-R2, tyrosine kinase and GLUT2, and positively with both LAIR and HAIR (*P*<0.01). On the contrary, adiponectin correlated positively with its receptors, tyrosine kinase and GLUT2, while negatively with insulin receptor isoforms (*P*<0.001), as shown in Table 4.

**Table 4:** Correlation coefficient (*r*) between HOMA-I and serum adiponectin with hepatic GLUT2, adiponectin receptors (Adipo- R1, R2), insulin receptor isoforms (LAIR, HAIR), and tyrosine kinase.

	GLUT2	Adipo-R1	Adipo-R2	LAIR	HAIR	Tyrosine kinase
**HOMA –I**	−0.645^**^ P = 0.004	−0678^**^ P = 0.002	−0807^**^ P = 0.000	0.649^**^ P = 0.004	0.840^**^ P = 0.000	−0.767^**^ P = 0.000
**Adiponectin**	0.824^**^ P = 0.000	0.942^**^ P = 0.000	0.893^**^ P = 0.000	−0.937^**^ P = 0.000	−0.960^**^ P = 0.000	0.912^**^ P = 0.000

Correlation was carried out in untreated and treated hyperglycemic animals.

## Discussion

To the best of the authors’ knowledge, this is the first study to examine the effect of topiramate on the hepatic insulin receptor isoforms, tyrosine kinase activity, adiponectin receptors and GLUT2, in a HFFD-induced insulin resistant model. The subchronic consumption of HFD, containing lard as the main constituent [23] along with 60% fructose, resulted in obesity/insulin resistance rat model without overt diabetic state. Such diet resembles the pathophysiologic scenario of what is known as ‘westernized diet’, which in addition to the sedentary life style is indicted for the widespread of obesity/insulin resistant cases nowadays.

The current model has upregulated insulin receptor isoforms, and decreased adiponectin receptors, tyrosine kinase activity, as well as GLUT2 level, besides interrupting serum glucose homeostasis, lipid profile, and adipocytokines. All these changes were positively modulated by the pre-existence of TPM, which was reported previously to enhance whole body insulin sensitivity by increasing glucose disposal, decreasing hepatic glucose output [11], [24] and inhibiting serum FFAs [11].

Insulin receptor exists in two isoforms that differ in the absence (Ex11^−^) or presence (Ex11^+^) of 12 amino acids sequence at the carboxylic terminus of the α-subunit. The difference is reflected on the binding affinity to insulin, where the first isoform, is less abundant in the liver and has higher affinity (HIR-A) to bind with insulin than HIR-B [25]. However, the expression of these isoforms is affected by several hormonal and metabolic factors [26], a fact that applies in the current study, where the insulin-resistant state induced high abundance of both isoforms. Although no available data on liver exists, yet previous works point to altered pattern of insulin receptor isotypes in skeletal muscle of pre-diabetic IR and NIDDM subjects [27], [28], and on isolated adipocytes of NIDDM patients [29], compared with normal insulin sensitive counterparts. Sbraccia et al. [30] showed also that expression of HIR-B correlates positively with hyperinsulinemia and negatively with insulin sensitivity in cases of insulin resistance.

The present model also inhibited tyrosine kinase activity, a finding that is corroborated by previous studies in cells of IR patients [31], and in liver biopsy [32] and skeletal muscles [33] of obese/T2D patients suggesting that this deficiency contribute to the I/R characteristics of both abnormalities. Thus, one may speculate that up-regulation of insulin receptor isoforms is a compensatory consequence to the model-induced hyperinsulinemia, and/or to the inactivated tyrosine kinase, which in turn impedes the proper autophosphorylation post-receptor cascades. From these results, it appears that a continuum of receptor and post-receptor defects are involved in the IR events in the liver of obese animals. Topiramate, on the other hand, down regulates insulin receptor isoforms possibly via maintaining insulin sensitivity, enhancing tyrosine kinase activity and improving glucose homeostasis, effects that were evidenced in this study.

Furthermore, the current model lowered the content of GLUT2, which is present mainly in the liver, while pre-treatment with TPM elevated it. This may be attributed to the improved insulin sensitivity and the corrected glucose panel, which maintain the GLUT2 gene expression. It was reported that portal hyperinsulinemia, in case of I/R, causes a sharp decrease in hepatic GLUT2 expression [34], [35], and proper glucose metabolism is required for the normal expression of the hepatic GLUT2 gene [36]. Although insulin does not affect hepatic glucose transport directly, yet the expression of some key enzymes, responsible for glucose metabolism, is under the influence of insulin [37], and so might GLUT2 [35].

The HFFD-induced hypoadiponectinemia and decreased expression of its receptors may be explained by the report of Lonardo et al. [2]. They stated that in clinical conditions such as T2D and obesity, peripheral IR correlates with hepatic IR and with hepatic steatosis (HS), an ailment that can be detected in rats fed even short-term high-fat diets, with the absence of obesity [38]. HS [39] and IR [40], [41] are linked with hypoadiponectinemia, and low levels of Adipo-R2, which is abundant mainly in the liver. HS is also suggested to cause hepatic IR by stimulating gluconeogenesis and activating other protein kinases that may interfere with tyrosine phosphorylation of insulin receptor substrate-1 (IRS-1) and IRS-2 and impair the ability of insulin to activate glycogen synthase and to translocate the glucose transporter GLUT-4 to the cell surface [2]. These results support the findings of the current study. Moreover, hypoadiponectinemia may result from obesity-induced IR in adipose tissue, especially the visceral ones [42]. The latter, in turn, mediates metabolic alterations in other peripheral tissues, especially liver and skeletal muscles [41]. Adiponectin, mediates its biological activity, at least partially, by binding to its receptors, and correlates to some extent to these receptors that were identified in 2003 [43]. Previous studies reported that decreased plasma adiponectin down regulates its receptors in the skeletal muscles of ob/ob mice [43], and that mice deficient in Adipo-R1 and R2 showed IR [44], reports that emphasize the present findings in the liver. On the other hand, the decreased expression of these receptors is thought to be responsible for the hypoadiponectinemia, which leads to insulin insensitivity; thus, the adiponectin/insulin resistance relationship resembles what is called “vicious cycle” [43].

The mechanisms by which adiponectin ameliorates insulin sensitivity include increased peripheral glucose uptake, muscle β-oxidation via the activation of AMP-activated protein kinase, suppressed hepatic glucose production, besides enhancing insulin-induced tyrosine phosphorylation of the IR with subsequent phosphorylation of IRS-1 [45]; nevertheless, the increased expression of the adiponectin receptors cannot be overruled. All these effects can, thus, rationalize the improved insulin sensitivity in the TPM treated groups, where this anticonvulsant boosted up adiponectin above normal [11], [46] and increased the abundance of its hepatic receptors, as presented in the current study. Topiramate also upregulates adiponectin gene expression [11], [41], which may be reflected on its receptors, as well. Moreover, in this study, a direct correlation was observed between the adiponectin receptors and the tyrosine kinase activity; hence, it is possible that activation of the hepatic tyrosine kinase may be mediated through the binding of adiponectin to its receptor(s), an assumption that remains to be proven.

Although TPM has been reported to decrease food intake and weight loss, yet it failed in this work to reduce rats’ weight gain induced by the HFFD, a result that confirms previous murine studies [15], [47], [48]. The possible factors of TPM-induced decrease in food intake, and consequently weight loss, did not play a role in our findings, since all comparisons made between HFFD+TPM-treated animals and pair-fed HFFD, regarding food intake were comparable between the two groups. However, controversial findings were reported in the literature. Clinically, some authors concluded that topiramate can significantly reduce body weight in obese people, with mild to moderate adverse effects [49]. Abo-Elmatty and Zaitone [50] also demonstrated that 2 months chronic treatment with topiramate (50 mg/kg), produced a significant weight loss in obese rats effects that could be referred to the chronic topiramate treatment. Topiramate has consistently decreased the efficacy of energy utilization in animal models, and effects on food consumption have varied with the model. These effects on energy efficiency may be mediated by stimulation of lipoprotein lipase in brown adipose tissue and skeletal muscle, thus increasing thermogenesis (10) or increasing the expression of uncoupling proteins 2 and 3 [49], thus directly decreasing the efficiency of energy utilization [51].

The anticonvulsant-mediated insulin sensitivity may correlate partly to the decrease in the VFW- and EFW−/body weight ratios, where visceral obesity [52] and white adipose tissue (WAT) are decisive steps in insulin resistance emerging. Epididymal fat tissue, akin to WAT, mediates chronic inflammatory reactions and releases macrophages, which sequentially discharge a pro-inflammatory adipokine, TNF-α; the latter enhances adipocytes to produce chemokines that aid in the recruitment of further macrophages in a ceaseless cycle [53]. TNF-α, elevated in the current study, induces IR by several mechanisms, including the down-regulation of adiponectin expression and inactivation of tyrosine kinase [54] and is responsible, partly, for hepatic fatty changes [2] characterized by elevated ALT. This enzyme is found primarily in the liver and is considered an indicator of hepatocellular health that can be compromised in case of obesity and IR [55], facts that coincide with the current results. It is also possible that high ALT reflects impaired insulin signaling, even if it is not associated with liver injury, where insulin inhibits gluconeogenic enzymes, including ALT [56]. Thus, the TPM-induced low ALT activity may be mediated by the improvement of insulin sensitivity, rather than by the lowering of TNF-α, which was significant yet subtle, as shown in Figure 2E.

Hepatic insulin resistance also enhances lipogenesis, events that can suggest a direct association between IR and fatty changes [57], along with raised ALT. In the present work, TPM hindered the model-induced lipid profile alterations, where it decreased levels of serum FFAs, TGs and TC. In a previous study, Liang et al. [57] reported that TPM regulates hepatic expression of genes involved in lipid metabolism, which could be part of the mechanisms by which TPM reduces plasma FFAs and TG levels in obese diabetic rodents. TPM reduces enzymes responsible for fatty acids synthesis and enhances those of fatty acid β-oxidation [57], as well as mitochondrial function [58], which accelerates the β-oxidation rate of fatty acids. Albeit scarce data are available regarding the effect of TPM on TC level, yet previous studies support our finding, where TC level was decreased in epileptic adults treated with TPM [59], and migraine patients receiving TPM [60] and alcoholic patients subjected to TPM [61]. However, the exact mechanism for this effect has not been clearly elucidated.

In conclusion, the results of the present study re-emphasize the vital role of the liver tissue in insulin resistance disorder, and confirm that the insulin-sensitizing effect of TPM involves mending hepatic alterations in obese/insulin resistant rats, besides its known correcting effect on hyperinsulinemia, dyslipidemia, and glucose intolerance.
